# Bone Marrow and Peripheral Blood AML Cells Are Highly Sensitive to CNDAC, the Active Form of Sapacitabine

**DOI:** 10.1155/2012/727683

**Published:** 2012-09-23

**Authors:** Sucheta Jagan, Laura A. Paganessi, Robin R. Frank, Parameswaran Venugopal, Melissa Larson, Kent W. Christopherson

**Affiliations:** ^1^Section of Bone Marrow Transplant and Cell Therapy, Rush University Medical Center, 1725 West Harrison Street, Chicago, IL 60612, USA; ^2^Rush University Cancer Center, 1725 West Harrison Street, Chicago, IL 60612, USA; ^3^Section of Hematology, Rush University Medical Center, 1725 West Harrison Street, Chicago, IL 60612, USA; ^4^Department of Anatomy and Cell Biology, Rush University Medical Center, 1725 West Harrison Street, Chicago, IL 60612, USA

## Abstract

Achieving improvements in survival and reducing relapse remains a challenge in acute myelogenous leukemia (AML) patients. This study evaluated the *in vitro* efficacy of the active form of novel agent sapacitabine, CNDAC, compared to current chemotherapeutic drugs Ara-C and mitoxantrone using two AML cell lines, HL-60 (promyelocytic) and THP-1 (monocytic), as well as bone marrow (BM) and peripheral blood (PB) cells collected from AML patients. Cell lines were exposed to compound for 3–6 days and primary cells for 4 days. The viability of primary cells was additionally evaluated 3, 7, and 31 days after removal of tested compound to determine the durability of the response. Our studies indicate that CNDAC and mitoxantrone have a greater impact on viability than ara-C in primary AML cells and AML cell lines. CNDAC is more effective at reducing viability and inducing apoptosis than ara-C at equivalent concentrations in the THP-1 cell line, which is defined as displaying resistance to ara-C. As sapacitabine has shown *in vivo* activity at clinically achievable doses, future studies are warranted to assess the potential for combining it with ara-C and/or mitoxantrone, with an emphasis on cells and patients insensitive to ara-C treatment.

## 1. Introduction

Acute myeloid leukemia (AML) therapy is continually challenged by high incidences of disease relapse and patient mortality. The overall 5-year survival rate for AML is 30–40% for patients >45 years and <10% for patients over 60 years [1]. However, the long-term event free survival rate of these patients is only 20–50% [2, 3]. The current AML therapy, “7 + 3” regimen with cytarabine (ara-C) and an anthracycline such as daunorubicin, idarubicin, or the synthetic anthracenedione mitoxantrone, has been the standard of care for decades. The nucleoside analog ara-C forms the backbone of AML treatment, either in low doses during induction therapy, or at high doses for maintenance after remission [4, 5]. Although high dose therapy has been shown to improve survival, 60–70% of patients relapse and eventually die due to disease progression [4, 6]. Moreover, there are patients who are nonresponsive to ara-C and many elderly AML patients cannot tolerate the regime and hence are not eligible for intensive chemotherapy. Novel therapeutic approaches are therefore required.

Nucleoside analogs, such as ara-C, represent a major group of antileukemic agents [5]. They are cell cycle-dependent cytotoxic agents that incorporate into the growing DNA strand forcing chain termination and inhibition of DNA synthesis. They are activated by the sequential addition of phosphates, firstly to the 5′ monophosphates form by the enzyme deoxycytidine kinase (dCK), and subsequently by other cellular enzymes which convert them into the di- and tri- phosphate forms, in preparation for incorporation into DNA [7]. Studies have indicated that resistance to nucleoside analogs mainly arise due to deamination by the enzyme cytidine deaminase (CDA) or due to the activity of the cytoplasmic enzyme 5′ nucleotidase, which dephosphorylates 5′ monophosphate products, opposing dCK activity [8]. Other resistance mechanisms include overexpression of transmembrane efflux pumps and reduced expression of topoisomerases [9]. Novel nucleoside analogs are mainly the result of minor structural modifications of existing drugs in an attempt to improve activity and suppress resistance [5].

Mitoxantrone is a synthetic anthracenedione that was developed as an analog to doxorubicin to reduce drug associated cardio-toxicity [10]. It is widely used in the treatment of previously untreated and relapsed AML patients [11]. Mitoxantrone is known to induce cell death by multiple mechanisms. At the molecular level, it markedly affects the activity of the enzyme topoisomerase II and causes DNA single and double strand breaks. The formation of a stable topoisomerase-DNA cleavable complex prevents rejoining of strand breaks. It intercalates stacked bases of DNA and can also bind to DNA via electrostatic cross-linking interactions. Oxidative activation of mitoxantrone generates free radicals that induce nonprotein associated strand breaks [11, 12]. At the cellular level, the drug is shown to be active as an immunosuppressant, affecting the activity of macrophages, T and B cells [10]. Therefore, mitoxantrone is active in both proliferating and nonproliferating cells. 2′-C-Cyano-2′-deoxy-1-*β*-d-arabino-pentofuranosylcytosine (CNDAC), the major metabolite of oral drug sapacitabine, is a nucleoside analog that is structurally related to ara-C and gemcitabine [13, 14]. The major difference is the addition of a cyano group replacing the 2′ hydrogen of the sugar moiety [14]. Ara-C and other nucleoside analogs induce cell cycle arrest in the S-phase. CNDAC, however, induces cell cycle arrest in the G2 phase following a delayed S phase [15]. Unlike ara-C, it does not cause chain termination at the site of incorporation. After additional elongation, the strong electrophilic property of the cyano group rearranges the nucleotide such that it lacks a free 3′ OH group [13, 16]. This resultant single strand break is minimally repaired by the cell's excision repair mechanism [17]. Replication results in a double strand break, eventually terminating DNA synthesis. In order to test the *in vitro* efficacy of CNDAC, this study compared CNDAC to conventional drugs, ara-C and mitoxantrone, in the promyelocytic cell line HL-60, which is known to be sensitive to ara-C, the monocytic cell line THP-1, known to be less sensitive to ara-C, and peripheral blood and bone marrow cells from 5 AML patients. 

## 2. Materials and Methods

### 2.1. Cell Lines

AML cell lines, HL-60 and THP-1, were obtained from ATCC (Manassas, VA) and cultured in RPMI 1640 medium (with phenol red) (Thermo Scientific Hyclone, Logan, UT) supplemented with 10% fetal bovine serum (Thermo Scientific Hyclone), 100 U/mL penicillin/100 *μ*g/mL streptomycin solution (Thermo Scientific Hyclone), and 2 mM L-Glutamine (Thermo Scientific Hyclone). Cells were cultured at 37°C, 5% CO_2_, and 100% humidity. Cells were in the logarithmic growth phase at the beginning of all experiments. 

M2-10B4 stromal cells were purchased from ATCC and cultured in RPMI 1640 media. Stromal layers were prepared by irradiating cells at 80 Gy and plating them on gelatinized 24 well plates at a concentration of 5 × 10^4^ cells/well. Wells were gelatinized by adding 0.1% gelatin in water (EMD Millipore, Billerica, MA) to each well and incubating for 6 hours. The M2-10B4 stromal layer was incubated at 37°C, 5% CO_2_, and 100% humidity for at least 24 hours before addition of AML cells.

### 2.2. Patient Samples

Peripheral blood (PB) and bone marrow (BM) specimens were obtained from 5 AML patients with informed consent on an IRB approved protocol in accordance with the Declaration of Helsinki. Of these patients, 3 were newly diagnosed and 2 had relapsed. The 2 relapsed patients had trisomy 13 and complex karyotypes, respectively. All other patients had normal cytogenetics. Total WBC counts of the patients ranged from 27.06 to 93.75 K/*μ*L with 56.6 to 98.5% blasts in the bone marrow. Additional clinical information is provided in Table 1. All specimens were collected prior to treatment. Peripheral blood was collected in heparinized vacutainer tubes and BM aspirates were collected in heparinized syringes. Mononuclear cell (MNC) fractions were obtained by density gradient centrifugation using Ficoll-Paque Plus (GE Healthcare, Piscataway, NJ). Cells were frozen and stored in liquid nitrogen and thawed before use. PB MNCs were treated with drug while in suspension, and BM MNCs were treated while in a coculture system with the mouse stromal cell line M2-10B4. Suspension medium was IMDM (Thermo Scientific Hyclone) supplemented with 20% FBS, 100 U/mL penicillin/100 *μ*g/mL streptomycin, 2 mM L-Glutamine, 100 ng/mL SCF (Stem Cell Technologies, Vancouver, Canada), and 50 ng/mL IL-3 (EMD Millipore, Billerica MA). Co-culture media was the same as suspension medium with the addition of 1 *μ*M hydrocortisone (Sigma Aldrich, St. Louis, MO).

### 2.3. Drugs, Treatment, and Culture Conditions

Cytarabine (ara-C) was purchased from Bedford Laboratories (Bedford, OH) and mitoxantrone from Mayne Pharma Limited (Mulgrave, Australia). CNDAC was kindly provided by Cyclacel Ltd. (Dundee, UK). Stock concentrations for ara-C (100 mM) and mitoxantrone (1 mM) were made in Dulbecco's Phosphate-Buffered Saline (DPBS) and stored at −80°C while CNDAC (100 mM) was dissolved in dimethylsulphoxide (DMSO) (Sigma Aldrich) and stored at −20°C. Working stocks of drugs were made in media.

Cell lines (HL-60 and THP-1) were treated in suspension in 48 well plates at seeding densities of 0.05 × 10^6^ cells/mL (low) or 0.5 × 10^6^ cells/mL (high). Cells were treated with ara-C or CNDAC at 0.5, 1, 2, 3, 4, 5, and 10 *μ*M and mitoxantrone at 0.0025, 0.005, 0.01, 0.02, 0.03, 0.04, and 0.05 *μ*M in triplicate at 37°C, 5% CO_2_, and 100% humidity. Appropriate untreated controls were included. Cells were analyzed 3, 4, 5, and 6 days posttreatment. 

1 × 10^6^ primary BM and PB cells were treated with 1 *μ*M (low), 10 *μ*M (medium), and 100 *μ*M (high) of ara-C or CNDAC or 0.005 *μ*M (low), 0.05 *μ*M (medium) and 0.5 *μ*M (high) mitoxantrone in 24 well plates at 37°C, 5% CO_2,_ and 100% humidity for 4 days. Appropriate untreated controls were included. Postdrug treatment, both PB and BM non-adherent cells were washed to remove compound, replated on M2-10B4 stromal layers, and reincubated at 37°C, 5% CO_2_, 100% humidity. Cells were analyzed immediately posttreatment and following 3, 7, and 31 days postdrug removal.

### 2.4. Alamar Blue Assay

The Alamar Blue assay was performed to determine drug IC_50_ values in AML cell lines. HL-60 and THP-1 cells were plated on 96 well flat bottom plates at 5 × 10^3^ cells/well. Cells were incubated at 37°C, 5% CO_2_, 100% humidity for 24 hours before addition of drugs. Cells were treated with ara-C, CNDAC and mitoxantrone in triplicate at 10 concentrations between 0.005 and 100 *μ*M. Plates were reincubated for 72 hours after addition of drugs. After 72 hours, Alamar Blue (AbD Serotec, Oxford, UK) was added to all wells at a final concentration of 10%, and plates were returned to the incubator for 8 hours before absorbance was read on the spectrometer (SpectraMax M5, Molecular Devices, Sunnyvale, CA) at wavelengths of 570 nm and 600 nm. Cell proliferation was determined by calculating the reduction of Alamar Blue. The equation used was as follows:

(1) %Reduction  in  Alamar  Blue  =[(O600  × A570)  −(O570  ×A600 )][(R570  ×N600 )  −  (R600 × N570)]  ×  100.

*O* and *R* are the molar extinction coefficients of Alamar Blue in its oxidized and reduced form, where *O*_600_ = 11726, *O*_570_ = 80586, *R*_570_ = 155677 and *R*_600_ = 14652. *N* is the absorbance of the negative control well. 

% Inhibition was calculated using the equation:

(2) [1−([Reduction(drug  conc.)−Reduction(negative  control  well)]Reduction(positive  control  well))]  ×100,

where the negative control was media + Alamar Blue, but no cells and the positive control was cells + Alamar Blue, but no drug.

### 2.5. Analysis of Viability

At each time point, cell lines and primary AML cells were assessed for overall viability by obtaining counts on a hemocytometer slide using trypan blue exclusion dye. Percentage live and dead cells in cell lines were calculated from the raw counts. For primary cells, Percentage of survival was calculated by dividing remaining live cells by the initial cell number.

### 2.6. Analysis of Apoptosis

Cells were washed twice in cold DPBS and resuspended in binding buffer containing appropriate volumes of 7AAD and AnnexinV and incubated in the dark for 15 minutes at room temperature. Excess antibody/dye was washed off, and data was acquired on the flow cytometer within 1 hour of staining. Cells were evaluated on a plot of 7-AAD versus AnnexinV. Cells that stained negative for 7-AAD and AnnexinV (7AAD^−^AnnexinV^−^) were considered live and non-apoptotic/healthy. Cells that stained positive only for AnnexinV (7AAD^−^AnnexinV^+^) were early apoptotic and those that stained positive for both 7-AAD and AnnexinV (7AAD^+^AnnexinV^+^) were late apoptotic or necrotic. The sum of cells in the 7-AAD^−^AnnexinV^+^ and 7-AAD^+^AnnexinV^+^ quadrants were total dead cells.

### 2.7. Statistical Analysis

Data are expressed as mean values ± SEM. Comparisons between drug treatments in cell lines were performed using the Students *t*-test, assuming equal variance. IC_50_ values of drugs in cell lines were determined from non-linear regression standard curve plots. In primary cells, the Mann-Whitney *U* Rank sum test was used to determine differences between groups, and the logrank test was used to compare curves. *P* ≤ 0.05 was considered significant. All statistical analyses were performed using SigmaPlot (Systat Software, Inc., Chicago, IL).

## 3. Results

### 3.1. Loss of Cell Proliferation

**Figure 1:**
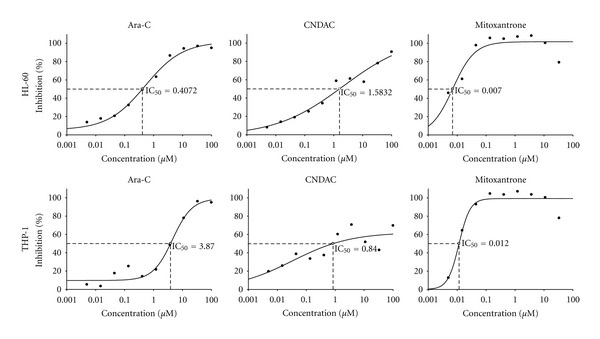
Standard curves and IC_50 _ values for cell lines ± drug treatment. Plots represent nonlinear regression standard curves and IC_50_ values of ara-C, CNDAC, and mitoxantrone for HL-60 (top) and THP-1 (bottom) cells as determined by Alamar Blue assay. Cells were drug-treated for 3 days and absorbance readings-taken 8 hours after addition of Alamar Blue. Inhibition (%) of drugs was calculated from reduction (%) of Alamar Blue. Experiments were run three times, in triplicate each time. Representative plots from one experiment are shown.

The IC_50_ values, defined as the half-maximal inhibitory concentration, for ara-C and CNDAC in HL-60 and THP-1 cells obtained by Alamar Blue assay (Figure 1) are comparable to other published data [13, 15, 18–22]. Similarities in the chemical structure and IC_50_ values of ara-C and CNDAC led us to test the same doses of both drugs in cell lines and primary cells. Mitoxantrone was active at much lower doses; therefore, cells were tested at 100-fold lower doses of the drug to see effects comparable to ara-C and CNDAC. Drug dilution ranges were chosen such that it covered IC_50_ values. The Alamar blue assay was deemed to be inappropriate for primary cells due to the assay's dependency on cell proliferation.

### 3.2. Sensitivity of Cell Lines to Ara-C, CNDAC, and Mitoxantrone

**Figure 2:**
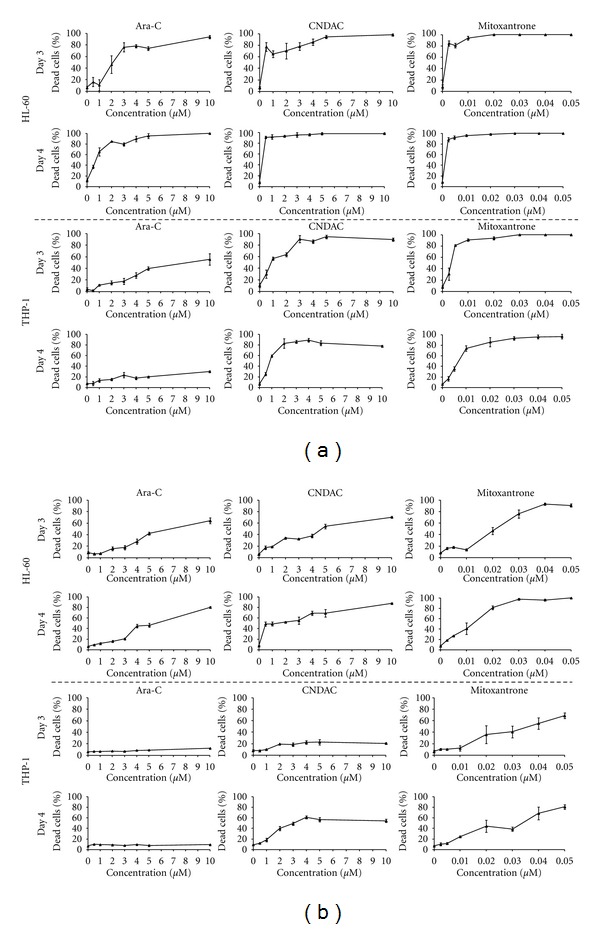
Viability of HL-60 and THP-1 cells ± drug treatment. Percentage of dead HL-60 (top) and THP-1 (bottom) cells plated at (a) 0.05 × 10^6^ cells/mL and (b) 0.5 × 10^6^ cells/mL. Cells were treated with ara-C, CNDAC, and mitoxantrone in triplicate; data shown is from drug treatments for 3 and 4 days.

To compare the effect of drugs at low and high cell proliferation rates, cell lines were plated at 2 seeding densities differing by 10-fold. HL-60 cells, plated at a high density, showed a dose response to ara-C (0.5 *μ*M to 10 *μ*M drug) with the % cell death ranging from 6.26 ± 1.39 to 63.8 ± 5.35 on day 3 (Figure 2(b)) and 8.32 ± 1.13 to 86.6 ± 6.36 on day 6 (*n* = 3). However, ara-C induced higher cell death in cells plated at the lower density with 16.2 ± 7.33 to 94.0 ± 2.82 % cell death on day 3 (Figure 2(a)) and 19.6 ± 2.88 to 100 ± 0.00 on day 6. Cell death induced by CNDAC at equivalent doses to ara-C, ranged from 17.3 ± 3.27% to 70.2 ± 0.84% on day 3 (Figure 2(b)) to 91.7 ± 1.02% to 95.9 ± 1.01% on day 6 for cells plated at the high density, and 77.4 ± 7.65% to 98.1 ± 1.75% on day 3 (Figure 2(a)) to 96.3 ± 2.9% to 100 ± 0.00% on day 6 for cells plated at the low density. At the higher cell density, although HL-60 cells showed a dose response to CNDAC on day 3, the % of cell death was not significant until day 4 at 0.5 *μ*M and 1 *μ*M of the drug (*P* ≤ 0.05, *n* = 3) (Figure 2(b)). At both cell densities, there was a significant increase in % of cell death between untreated and low dose drug-treated HL-60 cells on day 4 of drug treatment (*P* ≤ 0.05, *n* = 3). However, at lower seeding densities, equivalent concentrations of CNDAC were more effective than ara-C in inducing cell death (Figure 2(a)). The IC_50_ values of CNDAC, defined here as the concentration of drug required to induce 50% cell death were consistently lower than ara-C at all time points (Table 2).

THP-1 cells had a minimal response to ara-C at the high seeding density. Percentage of cell death at 10 *μ*M was 12.1 ± 0.28 on day 3 (Figure 2(b)) and 19.7 ± 2.31 on day 6 (*n* = 3). THP-1 cells had an overall low response to CNDAC on day 3, however, cell death was significantly higher than ara-C at doses >2 *μ*M (*P* ≤ 0.05, *n* = 3). There was also a significant increase in cell death for cells treated with ≥2 *μ*M CNDAC on day 4 as compared to day 3 (*P* ≤ 0.05, *n* = 3). The % of cell death for CNDAC-treated cells ranged from 39.9 ± 4.08 (2 *μ*M) to 54.6 ± 3.08 (10 *μ*M) on day 4 and 49.8 ± 1.55 to 86.9 ± 2.17 on day 6 (*n* = 3). Comparatively, at the lower seeding density, ara-C induced cell death at drug concentrations between 0.5 *μ*M and 10 *μ*M was highest on day 3—1.52 ± 1.67 % to 55.6 ± 9.87% (Figure 2(a)) and lowest on day 6—4.47 ± 1.04% to 6.84 ± 5.05%. At this cell density, THP-1 cells showed a dose response to CNDAC by day 3, with the % of cell death being significantly higher than ara-C at all doses tested (*P* ≤ 0.5, *n* = 3) (Figure 2(a)). IC_50_ values for CNDAC in THP-1 cells were lower than ara-C, regardless of seeding density (Table 2). 

The % of cell death from mitoxantrone ranged from 15.9 ± 1.89% (0.0025 *μ*M) to 90.8 ± 2.72 (0.05 *μ*M) on day 3 (Figure 2(b)) and 45.3 ± 4.67 to 100 ± 0.0% on day 6 for HL-60 cells plated at the higher cell density. THP-1 cells were comparatively less responsive to the drug with cell death ranging from 10.2 ± 1.48% to 68.8 ± 4.92% on day 3 (Figure 2(b)) to 19.7 ± 1.23% to 97.7 ± 1.21% on day 6. The % of cell death was significantly higher for cells treated with the lowest dose of mitoxantrone (0.0025 *μ*M) as compared to the untreated for HL-60 but not THP-1 cells (*P* ≤ 0.05, *n* = 3). At the lower seeding density, mitoxantrone was able to induce ≥84.9 ± 4.76% cell death in HL-60 cells on day 3 and only ≥30.7 ± 11.1% in THP-1 cells at doses of 0.0025 *μ*M and above (Figure 2(a)). However, both cell lines exhibited 100% cell death at doses > ss0.0025 *μ*M of mitoxantrone. 

**Figure 3:**
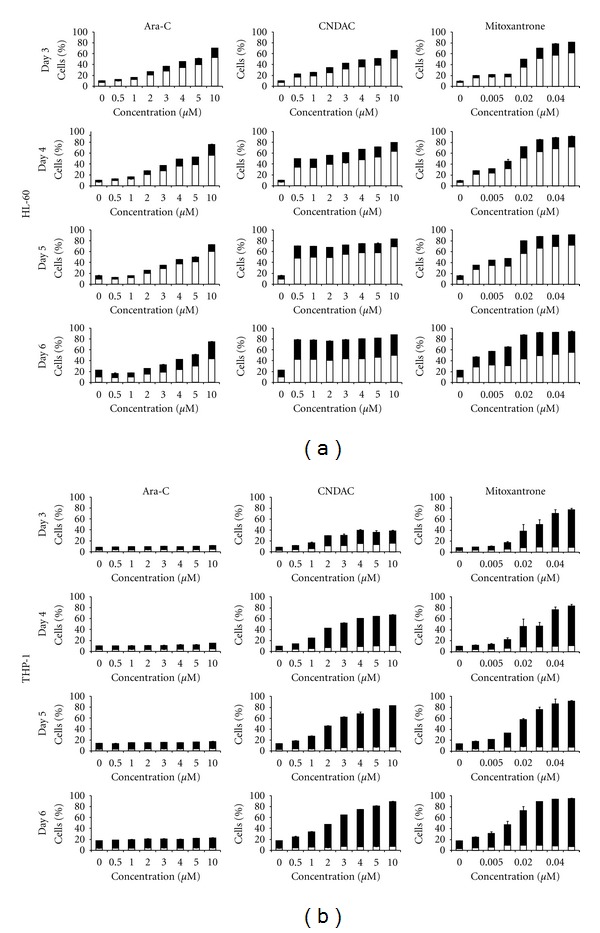
Apoptosis analysis of HL-60 and THP-1 cells. Plots represent distribution of □% early and ■% late apoptotic (a) HL-60 and (b) THP-1 cells treated with ara-C, CNDAC, or mitoxantrone as determined by 7-AAD/Annexin V staining. Cells were plated at 0.5 × 10^6^ cells/mL, in triplicate, and drug-treated for 3, 4, 5, and 6 days. The entire bar represents total dead cells.

Analysis of apoptosis was done for both cell lines at the high seeding density. Total dead cell numbers obtained from 7-AAD/Annexin V analysis complemented that from trypan blue counts. However, there was a difference in the distribution of early and late apoptotic events between the two cell lines. Of the total dead cells, the percent of late apoptotic/necrotic in HL-60 cells was ≤55.4 ± 0.76% and for THP-1 cells it was ≥58.4 ± 0.48% for all drug treatments and time points. This is indicative of the difference in the mode of cell death between the two cell types (Figure 3).

### 3.3. Sensitivity of Primary Cells from AML Patients to Ara-C, CNDAC and Mitoxantrone

Suspension and coculture systems for primary AML PB and BM MNCs were used during the first 4 days in order to mimic *in vivo* conditions during drug treatment. The co-culture system postdrug wash-out was used to provide additional support for expansion of both PB and BM cells that evaded drug effects. Assuming 100% survival at time zero of the experiments, the survival of PB MNCs increased to 115.6 ± 21.6% in the absence of drug when cultured in suspension during the first 4 days. Untreated BM MNCs in the co-culture system, however, had a slower growth rate with survival at 106.4 ± 18.9% after 4 days. By day 7 of culture (cells were washed and transferred to new stromal layers on day 4), the percent survival of PB and BM cells was 125.91 ± 38.7% and 130.91 ± 35.8% respectively. There was no further cell expansion beyond day 7 as survival was 130.83 ± 68.3% for PB and 129.24 ± 55.5% for BM cells on day 35 (31 days after replating).

**Figure 4:**
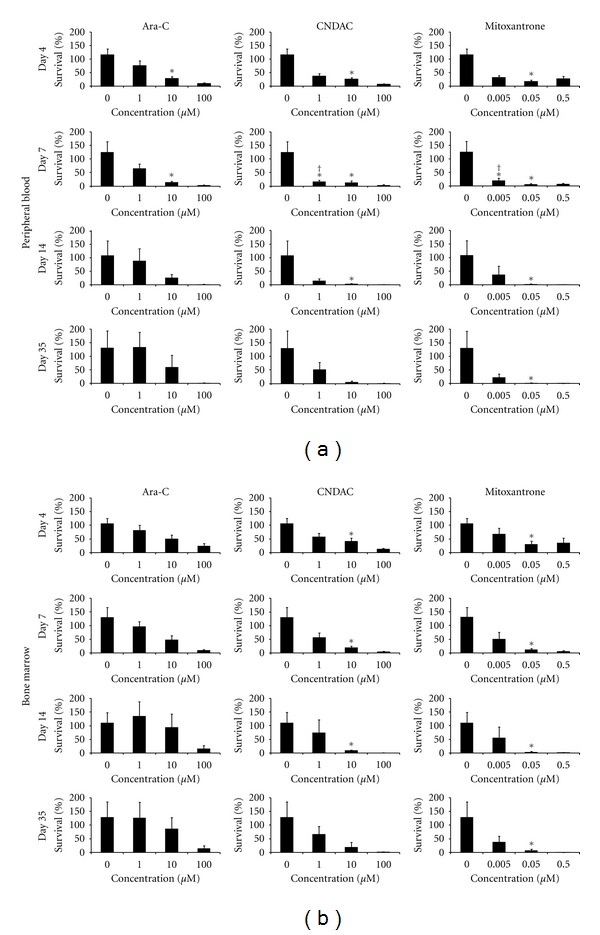
AML MNC ± drug treatment. Percentages survival (relative to number of cells plated) calculated from hemocytometer counts using trypan blue for (a) AML PB MNCs and (b) AML BM MNCs (*n* = 5 patients) treated with ara-C, CNDAC or mitoxantrone are shown. PB MNCs were drug-treated in suspension, while BM MNCs were treated in the presence of M2-10B4 stromal layers for 4 days (Day 4). Cells were then washed and replated on M2-10B4 stromal layers for post-drug wash-out analysis on days 7, 14, and 35. *denotes *P* ≤ 0.05 for particular drug concentrations compared with untreated, and ^†^denotes *P* ≤ 0.05 for 1 *μ*M CNDAC or 0.005  *μ*M mitoxantrone compared to 1 *μ*M ara-C.

**Figure 5:**
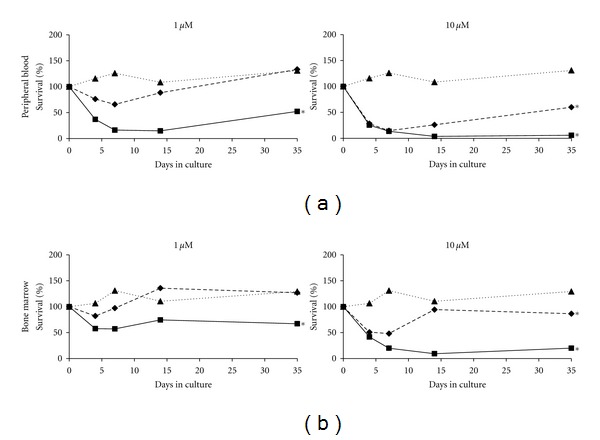
Growth of AML MNC ± drug treatment. Growth of untreated (…. dotted line), Ara-C treated (- - - dashed line) or CNDAC treated (— solid line) (a) PB and (b) BM AML cells from patients over the 35 day culture period, plotted as % survival relative to the number of cells initially plated. Drug treatment concentrations plotted are at 1 *μ*M and 10 *μ*M. *denotes *P* ≤ 0.05 as determined by the log rank test as compared to the survival curve of untreated cells.

Primary PB and BM MNCs were tested at the low, medium and high doses of ara-C, CNDAC, and mitoxantrone. The lack of availability of large cell numbers limited the number of doses tested. At any analysis time point, there was no significant difference in cell survival between untreated and low-dose ara-C (1 *μ*M) treated PB cells. However, 10 *μ*M ara-C induced a significant reduction in cell survival on days 4 and 7 when compared to the untreated (*P* ≤ 0.05, *n* = 5). Although not significant, the survival of PB cells trended lower than untreated (*P* = 0.056; *n* = 5) at 4 days post low-dose CNDAC treatment. By day 7, however, survival of CNDAC-treated cells at this dose was significantly lower than the untreated (*P* = 0.008, *n* = 5). Treatment with 10 *μ*M (medium dose) CNDAC resulted in a significant drop in cell survival compared to the untreated on days 4, 7, and 14 (*P* ≤ 0.05, *n* = 5). On day 35, cell survival still trended lower, but was not significant (*P* = 0.056; *n* = 5) (Figure 4(a)). Low dose (0.005 *μ*M) mitoxantrone-treated cells had significantly lower cell survival on days 4 and 7 (*P* = 0.016, *n* = 5) when compared to the untreated. Treatment with 0.05 *μ*M (medium dose) of mitoxantrone led to a significant loss in cell survival on all analysis days (4, 7, 14 and 35) (*P* ≤ 0.05, *n* = 5). At the high dose (100 or 0.5 *μ*M), all three drugs induced significant loss of survival of PB cells as compared to the untreated (*P* ≤ 0.05, *n* = 5) (Figure 4(a)). The overall survival of PB cells treated with low -dose CNDAC, and mitoxantrone was significantly lower than the untreated throughout the 35-day culture period (*P* ≤ 0.05, *n* = 5) (Figure 5(a)).

In the BM cells, the lowdose of either ara-C or CNDAC was not able to induce a significant loss in cell survival in comparison with untreated cultures at 4, 7, 14, or 35-day. However, lowdose mitoxantrone treated cells exhibited a downward trend in cell survival as compared to untreated (*P* = 0.056; *n* = 5). Unlike PB, the response of BM cells to 10 *μ*M ara-C was not statistically different from the untreated controls on days 4 and 7 (*P* = 0.056 and *P* = 0.69 resp., *n* = 5). The profile of medium-dose CNDAC (10 *μ*M) and mitoxantrone (0.05 *μ*M) treated cells when compared to untreated controls was similar to that seen in the PB (Figure 4(b)). Reduced viability of cells treated with low dose CNDAC and mitoxantrone as compared to the untreated cells over the entire culture period was maintained in the BM cells as it was in PB cells (*P* ≤ 0.05, *n* = 5) (Figure 5(b)).

Total cell survival in PB MNC, after 3 days of culture postdrug removal, was significantly lower for cells treated with 1 *μ*M CNDAC or 0.005 *μ*M mitoxantrone as compared to 1 *μ*M ara-C (*P* ≤ 0.05, *n* = 5) (Figure 4(a)). A similar trend was observed with the BM MNC, but did not reach significance (Figure 4(b)). Individual patient data indicated that CNDAC had an overall greater cytotoxic effect on cells as compared to ara-C irrespective of the differences in the mutational status of patients (clinical data not shown). 

## 4. Discussion

This study compared the cytotoxic effects of novel agent CNDAC to conventional agents, ara-C and mitoxantrone. The activity of ara-C and CNDAC is cell cycle dependent. Cell lines plated at low seeding densities have higher proliferation rates indicative of actively dividing cells thereby lowering IC_50_'s of the drugs. Conversely, at high seeding densities, cells have lower proliferation rates thus requiring higher doses of drug to achieve similar effects (Table 2). Regardless of seeding densities, HL-60 cells were more sensitive to CNDAC than ara-C. Significant cell death is induced by CNDAC at low doses (0.5 *μ*M), indicating that higher doses are unnecessary. A low effective dose is highly desired in the clinical setting, as low doses usually equate to less toxicity and meylosuppression in patients.

THP-1 cells are known to be less sensitive to ara-C due to high cytidine deaminase (CDA) activity, which deaminates ara-C into the inactive ara-U [7, 8, 18]. It is also shown to release the enzyme into the culture media thus deactivating the drug [18]. Ara-C was found to be active in these cells when plated at a low density albeit at very high concentrations (IC_50_ = 7.7 *μ*M). Higher cell death for ara-C-treated THP-1 cells on day 3 compared to day 6, when plated at the low density, can be explained by increasing amounts of CDA in the culture media resulting from active proliferation of cells. At the high seeding density, an IC_50_ for the drug in THP-1 cells was not achievable at the doses tested, perhaps due to complete inactivation of ara-C by CDA. THP-1 cells, however, responded to CNDAC with IC_50_ values ≤2.774 *μ*M, regardless of cell density (Table 2). The IC_50_ has been shown to be around 1 *μ*M in tumor cell lines [14] and mice [16]. Also, PK studies in humans have detected concentrations of CNDAC in plasma upwards of 0.25 *μ*M [2]. Together, these studies indicate that the data generated here is comparable to other studies and is likely clinically relevant. CNDAC is known to be a poor substrate for CDA, [16] thus explaining CNDAC's similar activity at both cell densities. Interestingly, CNDAC appears to have a delayed effect on THP-1 cells as a modest effect is seen on day 3 with a more robust effect seen on days 4–6 (Table 2 and Figure 2(a)). Mitoxantrone was largely unaffected by the seeding densities of cells. Although mitoxantrone has activity in both proliferating and nonproliferating cells, it is more active in cells in division than those in the latent phase [23]. This is evident in our experiments from the larger IC_50_ values seen at the higher cell densities and vice versa. Higher IC_50_ values for THP-1, compared to HL-60, suggest that THP-1 cells are slightly more resistant to mitoxantrone than HL-60 cells.

Stromal cells are often thought of as sanctuaries for primary leukemic cells. They produce cytokines and growth factors that protect cells in their niche and influence their proliferation, differentiation, and survival [24–26]. The immortalized human stromal cell line, HS-5, has been shown to protect AML cells from the toxic effects of ara-C [27, 28]. M2-10B4 feeder layers have been used in long-term culture initiating cell (LTC-IC) and cobblestone area forming cell (CAFC) assays and are known to provide antiapoptotic signals to both healthy and leukemic human and murine cells when cultured in contact [29–31]. The use of M2-10B4 co-culture systems to evaluate drug effects in primary cells from AML patients has not been reported previously. The culture system used in this study therefore provides a unique drug evaluation tool not limited to AML. 

In our experiments, while PB cells seemed more sensitive to effects of drugs, a fraction of BM cells was protected from toxic effects as they were treated with drug in the presence of stroma. At the low and middle doses, both PB and BM cells treated with ara-C seemed to grow out post drug removal with the support of the stromal cells (Figure 4). However, the outgrowth of BM cells was higher, indicating that stromal cells do not largely support the growth of PB cells. Contrastingly, there was a continuous loss of PB and BM MNCs treated with medium, and high doses of mitoxantrone as the cells cultured, suggestive of the longer effect of the drug even after washout. Additional studies which test the leukemic potential of the AML cells, that grow out postdug treatment, in immunodeficient mouse model systems are warranted. 

Unlike Ara-C treated cells, the residual cells, in both BM and PB after CNDAC-treatment, did not expand in culture. 2′- C-cyano- 2′, 3′-didehydro-2′,3′ -dideoxycytidine (CNddC), the chain terminating *β* elimination intermediate of CNDAC, is a poor substrate for DNA chain elongation and is responsible for inducing DNA single strand breaks. It is known to have a long shelf life in whole cells and its removal by the nucleotide repair (NER) mechanism is thought to be a slow process [17]. This unique mechanism therefore induces a prolonged effect on the survival of CNDAC treated cells. 

Our experiments have shown CNDAC to be more active than ara-C in both primary cells and cell lines. Sapacitabine is the palmitoyl derivative of CNDAC. The palmitoyl chain allows for oral absorption of the drug and protects the N4 amino group from deamination. A comparison of CNDAC and sapacitabine is presented by Serova et al. [15] The data presented here suggests that sapacitabine may be able to be administrated effectively to patients at lower doses than currently done; thus minimizing drug induced myelosuppression and toxicity to normal tissues. CNDAC has also been shown to be more active than ara-C *in vivo* in a P388 mouse leukemia model [32, 33]. Activity of CNDAC at low doses in THP-1 cells suggests its use as an alternative treatment option in patients resistant to ara-C. 

On the clinical front, sapacitabine has shown promising antileukemic activity. Phase I trials demonstrated the drug to be safe and active in the treatment of certain hematologic disorders (MDS and AML) and solid tumors alike [34]. It is also effective in AML and MDS patients with poor prognosis [2]. Sapacitabine is currently in Phase III trial for newly diagnosed AML patients who are 70 years or older and not a candidate for intensive induction therapy.

Future studies are warranted to assess the potential of combining CNDAC in the upfront setting of AML either simultaneously or in sequence with standard chemotherapy; particularly in patients who do not respond to ara-C-based therapies. There is a lifetime limit for the administration of mitoxantrone to patients as its toxicity is accumulative [35]. CNDAC, however, has no lifetime limit, making it a good candidate for maintenance therapy. In view of the low-toxicity profile, it is likely to be tolerated and a meaningful improvement in response can be expected.

## Figures and Tables

**Table 1:** Patient clinical characteristics.

Pt ID number	FAB	WBC (Th/ul)	Hb (g/dl)	Plt (Th/ul)	Cytogenetics	FISH	Flt-3 ITD	Flt-3 TKD	NPM	% Blasts PB	% Blasts BM
Normal range		4–10	13.5–17.5	150–450						−	≤5
005	M1	77	8.4	37	Trisomy 13	t/del(11)(q23) & del(20)(q12)	ND	ND	−	88.9	98.5
038	M4Eo	27.06	8.5	57	Inv 16	ND	−	−	ND	ND	71.8
042	ND	43.03	8.9	89	Normal	ND	+	−	+	53	56.6
045	M2	93.75	10.2	50	Normal	ND	−	+	−	21.5	99
059	ND	90.78	7.2	35	Complex	ND	ND	ND	ND	90	90

FAB: French-American-British classification; WBC: white blood cell; Hb: hemoglobin level; Plt: platelet level; FISH: fluorescence *in situ* hybridization; Flt-3: FMS-like tyrosine kinase 3; ITD: internal tandem duplications; TKD: tyrosine kinase domain; NPM: nucleophosmin; ND: not determined.

**Table 2:** IC_50_ values of Ara-C, CNDAC, and mitoxantrone for HL-60 and THP-1 cell lines plated at (A) 0.05 × 10^6^ cells/mL and (B) 0.5 × 10^6^ cells/mL.

IC_50_values (*μ*M)								
	(A)	0.05 × 10^6^ cells/mL			(B)	0.5 × 10^6^ cells/mL		
	Day	Ara-C	CNDAC	Mitoxantrone	Day	Ara-C	CNDAC	Mitoxantrone

HL-60	3	2.099	<0.5	<0.0025	3	6.157	5.356	0.021
	4	0.692	<0.5	<0.0025	4	5.031	1.149	0.011
	5	0.964	<0.5	<0.0025	5	5.503	<0.5	0.008
	6	1.929	<0.5	<0.0025	6	5.946	<0.5	0.003

THP-1	3	7.771	0.929	0.003	3	>10	>10	0.035
	4	>10	0.816	0.006	4	>10	2.774	0.030
	5	>10	1.105	0.011	5	>10	2.434	0.017
	6	>10	1.104	0.007	6	>10	2.095	0.012
